# A Rapid Method for Selecting Non-*Saccharomyces* Strains with a Low Ethanol Yield

**DOI:** 10.3390/microorganisms8050658

**Published:** 2020-05-01

**Authors:** Xiaolin Zhu, Yurena Navarro, Albert Mas, María-Jesús Torija, Gemma Beltran

**Affiliations:** Department of Biochemistry and Biotechnology, Faculty of Oenology, University Rovira i Virgili, c/Marcel·lí Domingo, 43007 Tarragona, Spain; xiaolin.zhu@estudiants.urv.cat (X.Z.); albert.mas@urv.cat (A.M.); mjesus.torija@urv.cat (M.-J.T.)

**Keywords:** alcoholic fermentation, wine yeast, sequential inoculation, ethanol reduction, *Metschnikowia pulcherrima*, *Torulaspora delbrueckii*, *Zygosaccharomyces bailii*

## Abstract

The alcohol content in wine has increased due to external factors in recent decades. In recent reports, some non-*Saccharomyces* yeast species have been confirmed to reduce ethanol during the alcoholic fermentation process. Thus, an efficient screening of non-*Saccharomyces* yeasts with low ethanol yield is required due to the broad diversity of these yeasts. In this study, we proposed a rapid method for selecting strains with a low ethanol yield from forty-five non-*Saccharomyces* yeasts belonging to eighteen species. Single fermentations were carried out for this rapid selection. Then, sequential fermentations in synthetic and natural must were conducted with the selected strains to confirm their capacity to reduce ethanol compared with that of *Saccharomyces cerevisiae*. The results showed that ten non-*Saccharomyces* strains were able to reduce the ethanol content, namely, *Hanseniaspora uvarum* (2), *Issatchenkia terricola* (1), *Metschnikowia pulcherrima* (2), *Lachancea thermotolerans* (1), *Saccharomycodes ludwigii* (1), *Torulaspora delbrueckii* (2), and *Zygosaccharomyces bailii* (1). Compared with *S. cerevisiae*, the ethanol reduction of the selected strains ranged from 0.29 to 1.39% (v/v). Sequential inoculations of *M. pulcherrima* (Mp51 and Mp FA) and *S. cerevisiae* reduced the highest concentration of ethanol by 1.17 to 1.39% (v/v) in synthetic or natural must. Second, sequential fermentations with *Z. bailii* (Zb43) and *T. delbrueckii* (Td Pt) performed in natural must yielded ethanol reductions of 1.02 and 0.84% (v/v), respectively.

## 1. Introduction

Global climate change has caused an increase in the alcohol content of wines in recent decades [1,2,3]. Specifically, global warming has accelerated maturation, increased the total soluble solids content and pH, and unbalanced the maturation of phenolic compounds and the increase in sugar concentration [2]. If grapes are harvested when phenolic compounds are mature, the grape must will have high concentration of sugars and low acidity, which produces wines with a high ethanol content. Otherwise, if the harvest occurs before that point, when sugar accumulation and pH are appropriate, wines will present a reduction in several characteristics (aroma, taste, and astringency) due to insufficient phenolic maturation. Regarding alcoholic fermentation, a high concentration of ethanol may lead to sluggish and stuck fermentations [4,5,6]. In addition, it can break the balance among acids, sugars, and tannins and develop unpleasant characteristics due to the enhancement of bitterness and burning sensation during tasting [7]. There are other reasons to achieve a lower ethanol content in wines, from their reduction in aromatic profile to the tax increase that will impact the final price of wines.

Previous studies about the reduction of alcohol in wines have focused on viticulture management, prefermentation, and postfermentation treatments and microbiological strategies during fermentation [8,9,10,11]. Specifically, from the point of viticulture management, a reduction in leaf area and removal of functional leaves were tested to reduce sugar accumulation, which could lead to a reduction in anthocyanins and soluble solids, delay maturity and significantly reduce the yield of grapes [12]. Another strategy for viticulture management is sequential harvesting, which aside from influencing phenolic maturity, could also affect the balance between fruity and vegetal aromas [13]. As a prefermentation treatment, García-Martín et al. [8] and Mihnea et al. [14] used membrane technologies, especially nanofiltration, to remove sugars from grape must. However, this method led to a reduction in wine color and flavor compounds. In addition, the removal of ethanol in wine was mainly considered during the postfermentation process. Aguera et al. [15] reported that removing 2% (v/v) ethanol had a significant effect on the concentration of volatile compounds, such as fusel alcohol and esters, which were reduced by 25% and 45%, respectively.

In recent years, microbiological strategies have garnered interest as alternatives to reduce ethanol concentrations [16,17,18]. For instance, the selection of evolved or modified strains from *Saccharomyces cerevisiae*, as well as low-ethanol producer strains from non-*Saccharomyces* yeasts, have been considered. In terms of *S. cerevisiae*, non-GMO strategies, such as evolutionary engineering, including experimental evolution under selective cultivation conditions or quantitative trait loci (QTL) mapping followed by breeding, have been used to improve industrial yeasts [19,20]. However, evolutionary engineering could affect some strain features under the conditions of industrial production and fermentation and lead to a distinct response of evolved strains to environmental factors that is different than that of ancestral strains [21]. The other strategy was genetic modification (GM), which has focused on changing the carbon metabolic conversion of sugar into other byproducts [16,22,23]. However, the application of GM methods in food and beverage production is forbidden due to poor public acceptance and regulations. Based on this limitation, screening non-*Saccharomyces* yeasts with alcohol-lowering abilities has become a consistent proposal to maintain wine quality and reduce the ethanol content [24,25,26,27,28,29]. The use of some non-*Saccharomyces* strains from *Candida*, *Hanseniaspora*, *Lachancea*, *Metschnikowia*, *Picha*, *Schizosaccharomyces*, *Starmerella*, and *Torulaspora* species has been shown to reduce ethanol in wines [18].

Non-*Saccharomyces* yeasts have been used in fermentations to reduce ethanol as a single or mixed inoculation. For example, *Candida sake* H14Cs reduced 2.4% (v/v) ethanol in natural must fermentations with a single inoculation [30]. Varela et al. [28] reported that, compared with *S. cerevisiae*, single fermentation by *Saccharomyces uvarum* AWRI2846 reduced the ethanol content by 1.7% (v/v), and coinoculated fermentation by *M. pulcherrima/S. cerevisiae* (10:1) reduced the ethanol content by 1% (v/v) in Merlot wines. Strains from *Hanseniaspora uvarum*, *Zygosaccharomyces sapae*, *Zygosaccharomyces bailii*, and *Zygosaccharomyces bisporus* species used as pure cultures in fermentations also showed a significant ethanol reduction in ethanol yield compared with *S. cerevisiae* [31]. However, the growth and metabolism of non-*Saccharomyces* yeasts will be affected by the presence of *S. cerevisiae*, especially in simultaneous fermentations [32,33]. Thus, sequential inoculation strategies, where *S. cerevisiae* is inoculated 24 or 48 h after the beginning of fermentation with non-*Saccharomyces* yeast, have been adopted by researchers and wine producers [34,35,36]. Englezos et al. [37] proposed a protocol to reduce ethanol based on the sequential fermentation of *S. bacillaris* and *S. cerevisiae*, showing higher ethanol reduction inoculating *S. cerevisiae* at 48 h than at 24 h.

In the present work, we proposed a rapid method to select non-*Saccharomyce*s strains with a low ethanol yield. The ethanol production and yield of 45 non-*Saccharomyces* yeasts, belonging to 18 species, were evaluated. After an initial screening on optimal medium for 3 days and on synthetic must for 48 h (to set the beginning of alcoholic fermentation), we reduced the number to 10 strains with a high potential to reduce the ethanol content. Afterwards, this ability was verified by complete fermentations in synthetic and natural must using sequential inoculations with a commercial *S. cerevisiae* wine yeast. In addition, all final samples were subjected to an in-depth chemical analysis to characterize the resulting wines.

## 2. Materials and Methods

### 2.1. Strains and Culture Conditions

One commercial wine yeast *Saccharomyces cerevisiae* (Lalvin QA23^®^, Lallemand Inc. Montreal, Canada, used as a control and referred to as Sc23) and forty-five non-*Saccharomyces* strains used in this study are listed in Table 1. Yeasts grew at 28 °C in YPD Agar (2% (w/v) glucose, 2% (w/v) yeast extract, 1% (w/v) peptone, and 1.7% (w/v) agar; Cultimed, Barcelona, Spain) and Wallerstein laboratory nutrient (WLN) agar (Becton, Dickinson and Company, Isère, France) from frozen stocks at −80 °C. Before starting fermentations, strains were identified at species level by PCR-RFLP analysis of 5.8S-ITS rDNA according to Esteve-Zarzoso et al. [38].

Propagation of strains was performed by picking a single colony from YPD plates. Strains grew in YPD liquid medium (2% (w/v) glucose, 2% (w/v) yeast extract, and 1% (w/v) peptone) for 24 h (Sc23) or 48 h (non-*Saccharomyces* strains) at 28 °C. After incubation, cells were counted in a Neubauer chamber (Leica Microsystems GMS QmbH, Leica, Germany), and 2 × 10^6^ cells/mL were inoculated into the appropriate fermentation medium. In all cases, the identity at the species level was confirmed by growth on differential WLN medium, and molecular identification by PCR-RFLP of 5.8S-ITS rDNA [38] was used to distinguish the non-*Saccharomyces* yeasts that presented similar morphological profiles with Sc23 on the WLN medium.

### 2.2. Fermentations

Three different media were used in fermentations, namely, YPD liquid medium, synthetic must (SM), and natural must (NM). SM was prepared according to Beltran et al. [40] (200 g/L sugars), and NM was obtained from Muscat grapes from Finca Experimental Mas dels Frares of Rovira i Virgili University (Constantí, Spain) during the 2019 vintage (219.2 g/L sugars, 4.52 g/L total acidity (as tartaric acid), 77.8 mg/L assimilable nitrogen, and pH 3.27). The nitrogen concentration in NM was corrected with diammonium phosphate (Panreac Quimica SA, Barcelona, Spain) until a final concentration of 240 mg N/L. Before the start of fermentation, dimethyl dicarbonate (0.2 mL/L) (ChemCruz^TM^ Biochemicals, Dallas, TX, USA) was added to NM, and kept at 4 °C for 24 h to eliminate the endogenous microorganisms. The absence of endogenous microorganisms was confirmed by plating a sample of the must on YPD Agar. Different fermentation procedures were performed in single and sequential fermentations. All fermentations were performed in triplicate, and single fermentations by Sc23 were used as a control.

In the first screening, strains were inoculated as single cultures in 5 mL YPD liquid medium in 12 mL tubes and incubated at 28 °C and 120 rpm for 3 days. Samples were taken daily to evaluate yeast growth, and after 3 days, extracellular media was kept to determine sugar and ethanol content. In the next step, strains were inoculated in 40 mL SM in 50 mL Falcon tubes, and fermentations were performed at 22 °C and 120 rpm and monitored over 48 h to evaluate yeast growth. Samples were taken at 48 h and centrifuged at 12000 rpm for 5 min, and the supernatant was kept at −20 °C until chemical compound analysis.

For sequential fermentations, experiments were carried out either in SM or NM. Non-*Saccharomyces* strains (2 × 10^6^ cells/mL) were used to start the fermentation, and 48 h later, Sc23 was inoculated (2 × 10^6^ cells/mL). Fermentations were conducted in 250 mL glass bottles with 230 mL of SM or NM (bottle caps were not tightly screwed in order to allow the release of CO_2_) and incubated at 22 °C with stirring at 120 rpm. SM and NM fermentations were monitored by evaluating yeast growth and must density which was determined with an electronic densitometer (Densito 30PX Portable Density Meter, Mettler Toledo, Hospitalet de Llobregat, Spain). The fermentation was considered finished when residual sugars were below 2 g/L, which was confirmed by enzymatic analysis in a Miura autoanalyzer (EE-MIURAONE Rev., I.S.E. S.r.l., Italy). Samples were centrifuged at 7800 rpm for 5 min, and the supernatants were frozen at −20 °C until analysis.

### 2.3. Population Dynamics

In single fermentation samples, the total population was assessed by microscope counting using a Neubauer chamber after 48 h of fermentation. Viability was also determined in sequential fermentations. Briefly, samples were serially diluted in sterilized Milli-Q water from a Milli-Q water purification system (Millipore S.A.S., Molsheim, France). The number of colony-forming units per milliliter (CFU/mL) was determined by plating 100 μL of three appropriately chosen dilutions on YPD, WLN, or lysine medium (11.75% (w/v) yeast carbon base, 2.5% (w/v) L-lysine monohydrochloride, and 20% (w/v) agar, Cultimed, Barcelona, Spain). Plates were incubated at 28 °C for 2 or 3 days.

### 2.4. Chemical Analysis

The glucose and ethanol contents of the samples from YPD cultures were determined with D-glucose and ethanol enzymatic bioanalysis kits (r-biopharm, Darmstadt, Germany), respectively. Residual sugars of samples at the end of fermentation in both SM and NM fermentations were quantified by D-glucose/D-fructose assays (Biosystems S.A., Barcelona, Spain).

Ethanol, glycerol, and organic acids (acetic acid, citric acid, malic acid, tartaric acid, lactic acid and succinic acid) in samples after 48 h of single fermentation and at the end of sequential fermentation and the sugars (glucose and fructose) after 48 h of single fermentation were determined by high-performance liquid chromatography (HPLC) using an Agilent 1100 HPLC (Agilent Technologies, Waldbronn, Germany) as previously described by Quirós et al. [41]. The HPLC was equipped with a Hi-Plex H column (300 mm × 7.7 mm) inside a 1260 MCT column compartment (Infinity II Multicolumn Thermostat) connected to MWD (G1365B multiwavelength detector) and RID detectors (1260 Infinity II refractive index detector) (Agilent Technologies, Waldbronn, Germany). The temperature of the column was maintained at 60 °C for a 30 min run time, and the mobile phase was 5 mM H_2_SO_4_ with a flow rate of 0.6 mL/min. The sample injection volume was 10 μL. Before injection, samples were filtered through 0.22 μm filters (Dominique Dutscher, Brumath, France). OpenLAB CDS (Agilent Technologies, Santa Clara, CA, USA) was used to analyze HPLC chromatographs.

### 2.5. Statistical Analysis

All graphs were generated using GraphPad Prism® version 8 (GraphPad Software, San Diego, CA, USA). The results are expressed as the mean ± standard deviation (SD). Statistically significant differences (one-way ANOVA) were analyzed by IBM SPSS Statistics version 23.0 (IBM, NY, USA). The ethanol yield was calculated with the formula “Ethanol yield (g/g) = ethanol production (g/L)/sugar consumption (g/L)”. Ethanol reduction was calculated by the formula “Δethanol (%, v/v) = Δethanol yield (g/g) × T sugars (g/L)/10 × 0.78924 (g/mL)”, where T sugars is the initial sugar concentration in the must and 0.78924 is the density of ethanol at room temperature.

## 3. Results

### 3.1. Rapid Screening of Non-Saccharomyces Strains with a Low Ethanol Yield

**Figure 1:**
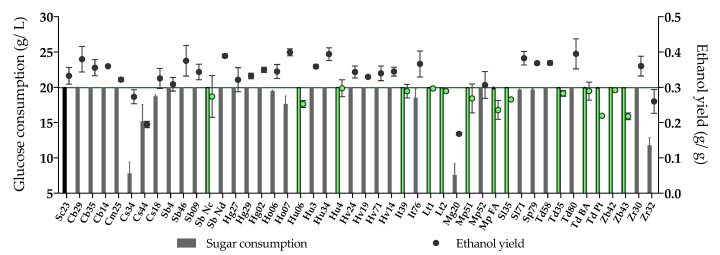
Glucose consumption (g/L) and ethanol yield (ethanol production (g/L)/sugar consumption (g/L), g/g) of 45 non-*Saccharomyces* yeasts and Sc23 (control yeast) after 3 days fermentation in YPD medium. The non-*Saccharomyces* yeasts selected for the next step are colored in green (glucose consumption > 19.90 g/L and ethanol yield ≤ 0.30 g/g). The value of the green line is 0.30 g/g (10% ethanol reduction of Sc23).

A first screening with forty-five non-*Saccharomyces* strains was performed under low sugar fermentation conditions (YPD medium), to evaluate the capacity of some yeast species and strains to consume sugars with a limited production of ethanol (fermentation vs. respiration capacity) (Figure 1, Table S1). *Saccharomyces cerevisiae* QA23 (Sc23), inoculated as a control, was able to consume all glucose (20 g/L) and produced 0.84% (v/v) ethanol, with an ethanol yield of 0.33 g ethanol/g glucose. The selection criteria for lower ethanol-producing yeast were established according to this result, taking into account their ability to consume glucose. Based on this, fourteen non-*Saccharomyces* strains were selected due to a high glucose consumption (> 19.90 g/L), and a lower ethanol production than that of the control with ethanol yields below 0.30 g/g (< 0.76%, v/v ethanol, 10% ethanol reduction compared with Sc23) (Figure 1). These strains belonged to the species *Hanseniaspora uvarum* (2), *Issatchenkia terricola* (1), *Lachancea thermotolerans (2), Metschnikowia pulcherrima* (2), *Saccharomycodes ludwigii* (1), *Starmerella bacillaris* (1), *Torulaspora delbrueckii* (3), and *Zygosaccharomyces bailii* (2).

**Figure 2:**
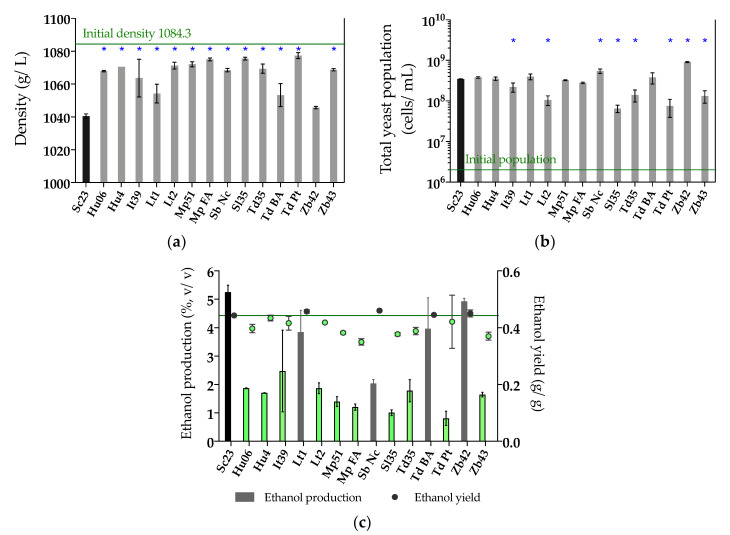
(**a**) Density (g/L); (**b**) Total yeast population (cells/mL); (**c**) Ethanol production (%, v/v) and ethanol yield (g/g) at 48 h of single fermentation in synthetic must. Non-*Saccharomyces* yeasts selected for the next step are colored in green, with the ethanol yield below that of Sc23 (0.44 g/g, the value of the green line in Figure (**c**)). Asterisk (*) means the significant difference compared with Sc23 (LSD, *p* < 0.05).

As non-*Saccharomyces* yeasts are commonly used in sequential fermentations, inoculating *S. cerevisiae* after 24-48 h, in the next step we analyzed the performance of the selected non-*Saccharomyces* strains during the first 48 h of fermentation. Therefore, we tested the 14 selected non-*Saccharomyces* strains on fermentation media (synthetic must) using Sc23 as a control. Must density, total yeast population, ethanol production, sugar consumption, and other main organic compounds were measured at 48 h of fermentation (Figure 2, Table S2). We observed that all selected strains were able to start fermentation in 48 h, consuming some of the sugars present in the must (with a corresponding decrease in must density, Figure 2a), although in a lesser amount than that of Sc23 (Table S2). The total yeast population showed that all strains were able to grow in fermentation media, and two of them, Sb Nc and Zb42, grew significantly higher than the control strain at 48 h (Figure 2b). The single fermentation with Sc23 was able to consume 47% (93.68 g/L) of total sugars in 48 h and produced the highest concentration of ethanol (5.26%, v/v), with an ethanol yield of 0.44 g/g (Table S2, Figure 2c). Most non-*Saccharomyces* strains consumed more glucose than fructose during 48 h, similar to the control Sc23 strain. However, three of the strains, Sb Nc, Sl35, and Zb43, consumed more fructose than glucose, and the two *H. uvarum* strains, Hu06 and Hu4, consumed equal quantities of glucose and fructose (Table S2). Ten out of 14 strains produced lower ethanol contents and lower ethanol yields than Sc23 (< 0.44 g/g), Hu06, Hu4, It39, Mp51, Mp FA, Lt2, Sl35, Td35, Td BA, and Zb43, and they were selected for subsequent experiments (Figure 2c).

### 3.2. Sequential Inoculation in Synthetic Must (SM) and Natural Must (NM)

To verify the ability of the 10 selected strains to reduce ethanol, sequential fermentations were performed. In the sequential fermentations, Sc23 was inoculated at 48 h in both SM and NM fermentations.

**Figure 3:**
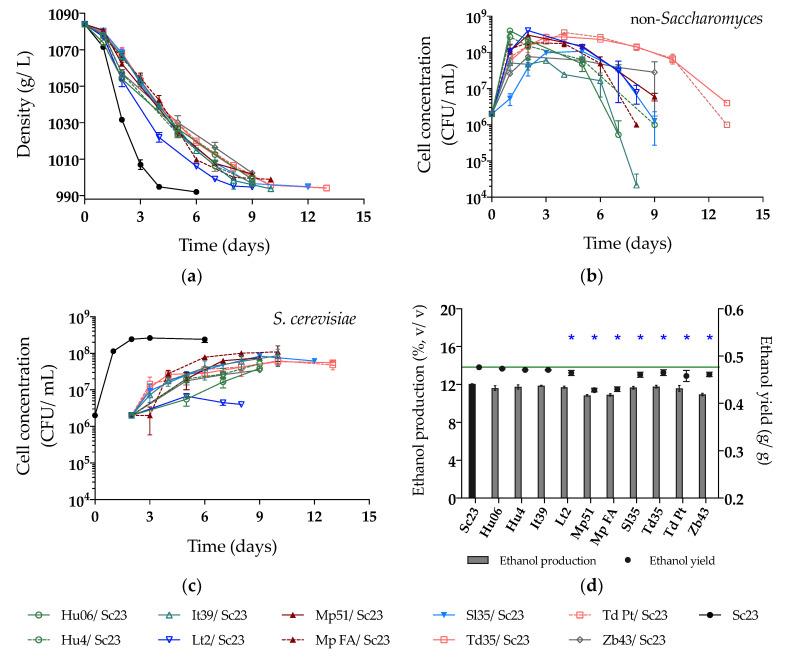
(**a**) Density (g/L); (**b**) Yeast population of non-*Saccharomyces*; (**c**) Yeast population of Sc23 and (**d**) Ethanol production (%, v/v) and yield (g/g) during sequential fermentations in synthetic must. The value of the green line in Figure (**d**) is 0.48 g/g (ethanol yield of Sc23). Asterisks (*) show the significant difference of ethanol yield compared with Sc23 (LSD, *p* < 0.05).

In the SM fermentation, Sc23 completed fermentation in 6 days, and the density of sequential fermentation trials with Lt2 showed the fastest reduction among the non-*Saccharomyces* strains. Nevertheless, more than 9 days were necessary to complete sequential fermentations by the other strains (Figure 3a). Interestingly, all non-*Saccharomyces* strains were detected during the fermentation process, with Hu06 and It39 being the strains with the fastest decrease in viability and Td Pt and Td35 maintaining relatively high viability until the end of fermentation (Figure 3b). Correspondingly, the Sc23 population reached a significant increase after inoculation at 48 h, with final viable populations between 3.5 × 10^7^ and 1.1 × 10^8^ CFU/mL, except in the Lt2/Sc23 fermentation, where Sc23 grew poorly (up to 6.7 × 10^6^ CFU/mL) (Figure 3c). Ethanol production decreased by 0.08 to 1.23% (v/v) in all sequential fermentations compared with that of the single fermentation by Sc23 (Figure 3d, Table 2), although this decrease was significant only with 7 of the non-*Saccharomyces* strains (Lt2, Mp51, Mp FA, Sl35, Td35, Td Pt, and Zb43). Higher concentrations of residual sugars were observed in the fermentation of Zb43/Sc23 and Hu06/Sc23. Our results (Table 2) showed that the sequential fermentations with *M. pulcherrima* strains Mp51/Sc23 and Mp FA/Sc23 had the highest ethanol reduction with the lowest ethanol yields (both are 0.43g/g compared to 0.48 g/g for Sc23).

**Figure 4:**
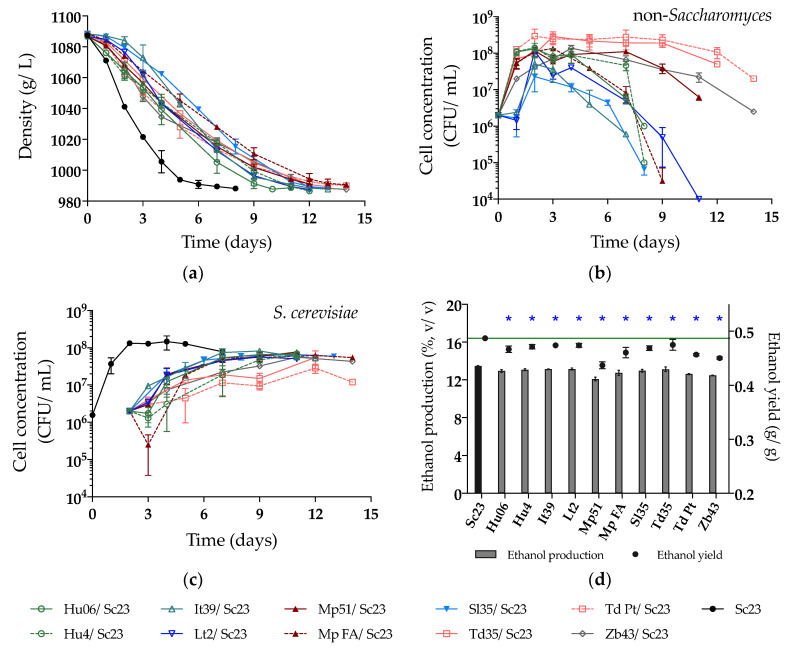
(**a**) Density (g/L); (**b**) Yeast population of non-*Saccharomyces*; (**c**) Yeast population of Sc23 and (**d**) Ethanol production (%, v/v) and yield (g/g) during sequential fermentations in natural must. The value of the green line in Figure (**d**) is 0.49 g/g (ethanol yield of Sc23). Asterisks (*) show the significant difference of ethanol yield compared with Sc23 (LSD, *p* < 0.05).

In the NM fermentation, all fermentations were delayed, probably due to the higher concentration of sugars in the natural must (219.2 g/L), especially fermentations that involved non-*Saccharomyces* strains, with Mp FA, Td Pt, and Zb43 taking the longest time, up to 14 days (Figure 4a). Noteworthy, the fermentation with Lt2/Sc23 was slower in NM, differing from the behavior observed in SM. In NM, the growth of Sc23 in sequential fermentations (Figure 4c) was higher than in SM (Figure 3c), and consequently, the growth of some non-*Saccharomyces* was hampered (Figure 4b). Only five non-*Saccharomyces* strains could be counted on WLN at the end of NM fermentations (Lt2, Mp51, Td35, Td Pt and Zb43) (Figure 4b), which were also the ones observed at the end of SM fermentations (Figure 3b). The ethanol production of all selected strains was reduced compared to the control fermentation with Sc23 (13.48%, v/v). The sequential fermentation by Mp51/Sc23 again showed the highest ethanol reduction, followed by Zb43/Sc23, Td Pt/Sc23, and Mp FA/Sc23 (Figure 4d, Table 2).

The production of glycerol differed significantly among the different sequential fermentations (Table 2), with Mp51/Sc23 and Mp FA/Sc23 fermentations having the highest glycerol levels in SM (10.3 and 9.83 g/L, respectively) and Lt2/Sc23 fermentations in NM (8.48 g/L). Indeed, the increase in glycerol of Mp FA/Sc23 and Lt/Sc23 fermentations, compared to single Sc23 fermentation, was significant both in SM and NM. The concentration of acetic acid remained under the recommended values for wines, below 0.35 g/L in SM and below 0.6 g/L in NM (the highest values were for Hu06 and Hu4 strains, 0.48 and 0.57 g/L, respectively). On the other hand, a significant increase in lactic acid was observed in the sequential fermentations performed with the Lt2 strain, both in SM and NM. Noteworthily, the concentration of succinic acid was significantly higher (1.35 g/L) in SM fermentation with Zb43.

## 4. Discussion

The selection of non-*Saccharomyces* yeasts to be used as fermentation starters, usually in combination with *S. cerevisiae*, has been mainly focused on improving the aromatic characteristics of wines [28,42] and reproducing the microbiota of vineyard or grapes [43]. In recent years, another reason for screening non-*Saccharomyces* yeasts has been the ability of some species to reduce ethanol content. Researchers have applied different combinations of non-*Saccharomyces* and *S. cerevisiae* yeasts to achieve this goal [24,27,44]. In this study, we focused on the selection of non-*Saccharomyces* yeasts with low ethanol yield by performing two short-term trials in 5 days. In the first selection step, we used YPD medium, which contains a low concentration of sugar, and analyzed ethanol yield and sugar consumption of the different strains. The metabolic characteristics of non-*Saccharomyces* yeasts will determine ethanol reduction, which implies that their metabolic footprints should be introduced before the inoculation of *S. cerevisiae* [26]. Therefore, the second selection step was performed in synthetic must for 48 h, in order to detect their ability to reduce ethanol before the inoculation of *S. cerevisiae*. With the selected strains, two sequential fermentation trials were performed, in synthetic and natural must, in which *S. cerevisiae* was inoculated after 48h. Simultaneous inoculations could reduce the contribution of non-*Saccharomyces* yeast in the fermentation process, and periods of longer than two days could jeopardize the imposition of *S. cerevisiae* and, as a consequence, the development of fermentation [33].

Regarding non-*Saccharomyces* screening strategies to achieve wines with low ethanol concentrations, Contreras et al. [25] used the fermentation of single yeast species in a defined medium for 4 days under anaerobic conditions to select strains from 50 non-*Saccharomyces* yeasts, followed by a second step with sequential fermentation for 7 days. After 11 days, eleven strains showed lower ethanol yields than *S. cerevisiae*. Another study reported by Contreras et al. [44] used a similar methodology over 11 days, and the difference was the use of semi-aerobic conditions of the initial fermentation. They selected seven strains out of 48 non-*Saccharomyces* yeasts with lower ethanol yield than *S. cerevisiae*. Quirós et al. [24] selected fifteen yeasts from 63 non-*Saccharomyces* strains by determining the respiratory quotient under fully aerobic conditions in 4 days, followed by the performance of selected strains in synthetic must for 4 days. However, after 8 days of analysis, several of the selected strains showed a higher ethanol yield than that of *S. cerevisiae*. Thus, compared to previous selection trials, the screening process applied in our study was equally rapid, but the pre-selection of strains was more reliable, and we included an important screening criterion to be considered, that is, the selected non-*Saccharomyces* yeasts were able to finish fermentations under low-sugar conditions.

The ethanol yields of *S. cerevisiae* in YPD medium (0.33 g/g) were lower than those in semi-anaerobic fermentative conditions (approximately 0.48 g/g), which agrees with the results of Quirós et al. [24] in fully aerobic conditions, where the ethanol yield of *S. cerevisiae* was approximately 0.25–0.30 g/g. Instead, when fermentative conditions in synthetic or natural must were used, the ethanol yields for *S. cerevisiae* were close to the expected values (i.e., 0.44–0.48 g/g) [25]. The differences could be due to the importance of respiratory metabolism in YPD medium, where the sugar concentration was low (20 g/L), whereas in synthetic or natural must, with high sugar concentrations (≥ 200 g/L), glucose repression occurred [45]. 

In general, most non-*Saccharomyces* yeasts present weak fermentation capacity and grow slower than *S. cerevisiae* [46,47]. Similar results were observed in the current study, where all non-*Saccharomyces* yeasts started fermentations slower than Sc23, and eight of the strains had poorer growth than Sc23 during the first 48 h. In the present work, Sc23 consumed almost half of the sugars at 48 h and presented the highest sugar consumption among all fermentations. This is supported by previous studies in which different non-*Saccharomyces* strains, *M. pulcherrima*, *S. bombicola*, *H. uvarum*, *T. delbrueckii*, and *Z. bailii* consumed less sugar than *S. cerevisiae* in a single fermentation before 48 h [24,34,44]. On the other hand, four of the strains had faster growth and higher ethanol yields than Sc23 (Lt1, Sb Nc, Td BA, and Zb42), and three of them (Lt1, Td BA, and Zb42) also had higher sugar consumption at 48 h in SM. Thus, during alcoholic fermentation in synthetic must, growth seemed to be positively correlated with sugar consumption and ethanol yield. In fully aerobic conditions, Quirós et al. [24] also observed a positive correlation between ethanol yield and sugar consumption in non-*Saccharomyces* strains but a negative correlation with biomass, which may be due to the higher growth capacity in respiratory conditions.

After the proposed screening, we demonstrated that the ten selected non-*Saccharomyces* yeasts reduced the ethanol content, in both synthetic and natural musts, by sequential fermentations. Therefore, the strategy of two short-term trials in 5 days to select the non-*Saccharomyces* strains was appropriate, as the ethanol reduction was confirmed for most strains. Moreover, the timing of *S. cerevisiae* inoculation in the sequential fermentations (48 h) was also appropriate, as most non-*Saccharomyces* species could persist until mid-end of the fermentation, showing an impact on the ethanol content and the final product. 

Non-*Saccharomyces* yeasts lose viability during alcoholic fermentation and are soon replaced by *S. cerevisiae*. This may be due to several factors, such as low resistance to ethanol [48], nutrient competition [33,49,50], or microbial interactions, either by cell-to-cell contact [51,52,53] or the secretion of antimicrobial compounds by different yeasts (mainly *S. cerevisiae*) [54,55,56]. The populations of Lt2, Mp51, Td Pt, Td35, and Zb43 were found viable until the end of fermentation (cultivating on WLN), although they showed different performance in SM and NM fermentations. This persistence seems to be inconsistent with previous studies that claimed that most non-*Saccharomyces* species cannot tolerate ethanol concentrations above 5–7% (v/v) [26,52,55]. However, we have recently shown that *L. thermotolerans* and *T. delbrueckii*, used as a single culture, are able to finish a fermentation with 200 g/L of sugars, producing up to 9–10% (v/v) of ethanol [57]. Even if the presence of *S. cerevisiae* in mixed fermentations can induce the death of other yeast species [58], other studies have shown that the presence of both *Saccharomyces* and non-*Saccharomyces* yeasts increased the persistence of non-*Saccharomyces* yeasts during the fermentation process [47,59]. Indeed, interactions between *Saccharomyces* and non-*Saccharomyces* wine yeasts have an effect not only on the persistence of the non-*Saccharomyces* yeasts but also on the behavior of the *Saccharomyces* wine strains [32]. Thus, the survival of these non-*Saccharomyces* yeasts until the end of fermentation in the current study might be a result of possible synergistic interactions between yeasts, and also due to their tolerance to a higher alcohol content, although this fact needs to be confirmed by further research.

Our results also showed that yeast performance and survival was influenced by the type of must (SM and NM), which could be due to the different nutrient composition. Indeed, we have previously observed that different sugar and nitrogen concentrations on the must have a clear effect on the evolution of mixed fermentations done with *H. uvarum*, *S. bacillaris*, and *T. delbrueckii* species, both on sugar consumption and population dynamics [33]. Another study showed different sugar consumption profiles between Chardonnay and Shiraz grape must (with 240 and 210 g/L sugars, respectively) in mixed fermentations with *M. pulcherrima/S. cerevisiae* [27]. Moreover, in a previous study we observed that changes in the concentration of some fermentation metabolites had an effect on the culturability of some non-*Saccharomyces* strains (*H. uvarum*, *S. bacillaris* and *M. pulcherrima*) when used in mixed fermentations [53]. 

In the present work, Mp51/Sc23 fermentation demonstrated the highest ethanol reduction of 1.23% (v/v) in SM and 1.39% (v/v) in NM. The other strain belonging to the *M. pulcherrima* species, Mp FA, reduced the ethanol content by 1.17% (v/v) in SM fermentation. Similar to previous studies, *M. pulcherrima* has been recognized as a strain with a relatively high capacity to reduce ethanol in sequential fermentation with *S. cerevisiae* and had exhibited ethanol reductions by 0.9 to 3.6% (v/v) [25,60,61]. In addition, fermentation by Zb43/Sc23 and Td Pt/Sc23 reduced the ethanol content by 1.02 and 0.84% (v/v) in NM fermentation, respectively. In agreement with previous research, *Z. bailli* and *T. delbrueckii* in sequential fermentation reduced the ethanol content by 1.0 to 1.6% (v/v) [31,44].

During the fermentation process, the reduction of the ethanol concentration by non-*Saccharomyces* yeasts could be explained not only by their greater accumulation of yeast biomass but also by other byproducts produced after consuming sugars [26]. Under sufficient oxygen availability, carbon from sugar metabolism can be diverted towards organic acids and glycerol, resulting in low ethanol production [62,63]. As the present study aims to be a method for screening non-*Saccharomyces*, we only evaluated the concentration of main by-products after alcoholic fermentation. Interestingly, the content of byproducts was influenced by the type of must used. In the current study, the highest concentration of glycerol was achieved in fermentations with Mp51 and Mp FA but only in SM. Furthermore, the production of glycerol in Mp51/Sc23 fermentation was affected by the type of must, as no significant increase was observed in NM fermentation, even if the highest ethanol reduction was obtained in this condition. As discussed before, the different nutrient composition of the must could affect the viability and metabolism of some non-*Saccharomyces* strains [27,53]. Thus, the current study reveals that the glycerol production should not be the only metabolic pathway to reduce ethanol content. On the contrary, the highest concentration of glycerol was observed in NM fermentation with Lt2. This was consistent with the results from Gobbi et al. [61] when fermentations with *L. thermotolerans* generated higher concentrations of glycerol (more than 7 g/L) in natural must. Associated with the overproduction of glycerol caused by ethanol reduction, the concentration of acetic acid might be increased, mainly in aerobic conditions [64,65]. However, in the present work, performed in semi-anaerobic conditions, the fermentation with Mp FA/Sc23 in SM and NM significantly reduced the concentration of acetic acid, although increased the glycerol content, when achieving an ethanol reduction. The same performance was also observed in sequential fermentations with Sl35 and Zb43 in SM. These results supported those of Morales et al. [65], where *M. pulcherrima* was able to reduce the concentration of acetic acid while increased glycerol and reduced ethanol content in mixed fermentations, compared with single *S. cerevisiae* inoculation. In the current study, the concentration of acetic acid remained below 0.8 g/L, considered the level when acetic acid may confer unpleasant acidic taste to wine [46]. Nevertheless, our results showed that both *H. uvarum* strains (Hu4 and Hu06) have significantly increased the acetic acid content in NM wines, confirming its higher production of negative byproducts and its poor oenological performance [66]. Previous studies have shown that in *T. delbrueckii* and *H. uvarum* species acetic acid production was unrelated to ethanol formation, being *T. delbruecki* a low and constant acetic acid producer, and *H. uvarum* a high acetic acid producer species [66]. Additionally, fermentation by *L. thermotolerans* (Lt2/Sc23) produced the highest concentration of lactic acid in SM and NM fermentations, especially in NM fermentation. Strains of *L. thermotolerans* are frequently used for the acidification of low-acidity wines due to their ability of producing lactic acid during wine fermentations [67,68]. Our results also agreed with Binati et al. [69], who reported that sequential fermentation with *L. thermotolerans* followed by inoculation of *S. cerevisiae* at 48 h produced a high concentration of lactic acid and reduced the ethanol content by 0.35% (v/v) in Pinot Grigio must. In our study, Lt2 reduced approximately 0.35% (v/v) ethanol in SM and NM fermentations. The highest content of succinic acid was produced in SM fermentation with Zb43. Likewise, sequential fermentation by *Z. bailli* increased the concentration of succinic acid in defined grape must [44].

In conclusion, this was a rapid method for screening yeasts with low ethanol yields. *M. pulcherrima* Mp51 and Mp FA are two appropriate wine yeasts for reducing ethanol in sequential fermentation trials. The potential of *Z. bailii* Zb43 and *T. delbrueckii* Td Pt to reduce ethanol concentrations needs to be explored. In addition, a complete analysis of the aromatic compounds should be analyzed to determine the impact of those sequential fermentations and ethanol reduction on wine quality and flavor. Thus, further research should focus on optimizing the inoculation time of non-*Saccharomyces* strains in sequential fermentation, as well as on the chemical and sensory analysis of the resulting wines. However, the application at the industrial scale is still a challenge to be addressed in the future.

## Figures and Tables

**Table 1:** Yeast strains used in this study (CECT, Spanish Type Culture Collection; URV, our group yeast collection, some of them isolated in Priorat Appelation of origin [39]; UdlaR, Universidad de la República yeast collection, Uruguay; Agrovin S.A, Ciudad Real, Spain; Lallemand, Lallemand Inc. Montreal, Canada).

Yeast Species	Strain Designation	Collections	Isolation Source	Abbreviations in This Paper
*Saccharomyces cerevisiae*	QA23	Lallemand	Commercial	Sc23
*Candida boidinii*	10029	CECT	Milk	Cb29
	10035	CECT	Frass on *Amygdalus* *communis*	Cb35
	1014 ^T^	CECT	Tanning fluid	Cb14
*Candida mesenterica*	1025	CECT	Brewery	Cm25
*Candida sake*	10034	CECT	Feces of sheep	Cs34
	1044	CECT	Lambic beer	Cs44
*Candida stellata*	11918^T^	CECT	Wine grape	Cs18
*Starmerella bacillaris*	4	URV	Grape must (Priorat)	Sb4
	11046	CECT	Grape juice	Sb46
	11109	CECT	Wine	Sb09
	NS c	URV	Grape must	Sb Nc
	NS d	URV	Grape must	Sb Nd
*Hanseniaspora guilliermondii*	11027	CECT	Grape must	Hg27
	11029 ^T^	CECT	Infected nail	Hg19
	11102	CECT	Grape juice	Hg02
*Hanseniaspora osmophila*	11206	CECT	Ripe Riesling grape	Ho06
	11207	CECT	Grape	Ho07
*Hanseniaspora uvarum*	11106	CECT	Wine grape	Hu06
	13130	CECT	Grape must (Priorat)	Hu4
	3	URV	Grape must (Priorat)	Hu3
	34	URV	Grape must (Priorat)	Hu34
*Hanseniaspora vineae*	11.24	UdlaR	Grapes (Uruguay)	Hv24
	12.219	UdlaR	Grapes (Uruguay)	Hv19
	13714	CECT	Nd^a^	Hv14
	1471	CECT	Grape juice	Hv71
*Issatchenkia terricola*	11139	CECT	Dregs of pressed grapes	It39
	11176	CECT	Soil	It76
*Lachancea thermotolerans*	1	Agrovin	Nd ^a^	Lt1
	2	Agrovin	Nd ^a^	Lt2
*Meyerozyma guilliermondii*	1020	CECT	Nd ^a^	Mg20
*Metschnikowia pulcherrima*	51	URV	Grape must (Priorat)	Mp51
	52	URV	Grape must (Priorat)	Mp52
	FLAVIA	Lallemand	Commercial	Mp FA
*Saccharomycodes ludwigii*	1235 ^T^	CECT	Nd ^a^	Sl35
	1371	CECT	Nd ^a^	Sl71
*Schizosaccharomyces pombe*	1379	CECT	Nd ^a^	Sp79
*Torulaspora delbrueckii*	10558	CECT	White wine	Td58
	13135	CECT	Grape must (Priorat)	Td35
	1880	CECT	Wine of Airen grape	Td80
	Priorat	URV	Grape must (Priorat)	Td Pt
	BIODIVA	Lallemand	Commercial	Td BA
*Zygosaccharomyces bailii*	11042	CECT	Grape must	Zb42
	11043	CECT	Cloudy wine	Zb43
*Zygosaccharomyces rouxii*	1230	CECT	Honey	Zr30
	1232	CECT	Concentrate must	Zr32

^T^ presents Type strain; ^a^ presents No description.

**Table 2:** Analysis of sugars, ethanol, organic acids, and glycerol from samples at the end of sequential fermentations.

	Residual Sugar	Sugar Consumption	Ethanol Production	Ethanol Yield	Ethanol Reduction	Succinic Acid	Lactic Acid	Acetic Acid	Glycerol
	(g/L)	(g/L)	% (v/v)	(g/g)	% (v/v)	(g/L)	(g/L)	(g/L)	(g/L)
Synthetic must fermentation									
Sc23	0.12 ± 0.10	199.88 ± 0.10	12.07 ± 0.02	0.48 ± 0	0 ± 0	0.56 ± 0.04	0.23 ± 0	0.30 ± 0.03	5.76 ± 0.16
Hu06	6.24 ± 3.83 *	193.76 ± 3.83 *	11.62 ± 0.25 *	0.47 ± 0	0.08 ± 0.02	0.54 ± 0.08	0.18 ± 0.01	0.24 ± 0.04	6.60 ± 0.22
Hu4	3 ± 3.94	197 ± 3.94	11.75 ± 0.23	0.47 ± 0	0.14 ± 0.01	0.41 ± 0.01	0.16 ± 0.01	0.28 ± 0.02	6.90 ± 0.03
It39	0.71 ± 1	199.29 ± 1	11.89 ± 0.03	0.47 ± 0	0.15 ± 0.03	0.46 ± 0.06	0.23 ± 0.02	0.33 ± 0.01	7.65 ± 0.84 *
Lt2	0.82 ± 0.43	199.18 ± 0.43	11.72 ± 0.11	0.46 ± 0.01 *	0.31 ± 0.14 *	0.56 ± 0	0.55 ± 0.04 *	0.27 ± 0.11	7.44 ± 0.33 *
Mp51	0.03 ± 0.04	199.97 ± 0.04	10.85 ± 0.09 *	0.43 ± 0 *	1.23 ± 0.10 *	0.58 ± 0.01	0.21 ± 0.03	0.28 ± 0.04	10.30 ± 0.45 *
Mp FA	0 ± 0	200 ± 0	10.90 ± 0.12 *	0.43 ± 0 *	1.17 ± 0.12 *	0.59 ± 0.03	0.16 ± 0.01	0.16 ± 0.06 *	9.83 ± 0.67 *
Sl35	0.12 ± 0.06	199.88 ± 0.06	11.67 ± 0.14 *	0.46 ± 0.01 *	0.40 ± 0.14 *	0.65 ± 0.01	0.22 ± 0.11	0.17 ± 0.01 *	7.76 ± 0.93 *
Td35	0.24 ± 0.05	199.77 ± 0.05	11.77 ± 0.15	0.47 ± 0.01 *	0.29 ± 0.15 *	0.53 ± 0.16	0.16 ± 0.04	0.26 ± 0.04	5.31 ± 0.12
Td Pt	0.40 ± 0.15	199.61 ± 0.15	11.59 ± 0.30 *	0.46 ± 0.01 *	0.47 ± 0.29 *	0.53 ± 0.02	0.37 ± 0.05 *	0.24 ± 0.05	5.36 ± 0.10
Zb43	12.62 ± 2.58 *	187.38 ± 2.58 *	10.95 ± 0.12 *	0.46 ± 0 *	0.39 ± 0.11 *	1.35 ± 0.52 *	0.31 ± 0.12	0.12 ± 0.01 *	8.61 ± 0.86 *
Natural must fermentation									
Sc23	0.76 ± 0.10	218.42 ± 0.10	13.48 ± 0.03	0.49 ± 0	0 ± 0	0.77 ± 0.08	0.16 ± 0.08	0.31 ± 0.04	5.56 ± 0.06
Hu06	0.41 ± 0.16	218.76 ± 0.16	12.94 ± 0.16 *	0.47 ± 0.01 *	0.56 ± 0.17 *	0.73 ± 0.04	0.15 ± 0.01	0.48 ± 0.17 *	6.60 ± 0.75 *
Hu4	0.19 ± 0.14	218.98 ± 0.14	13.08 ± 0.11 *	0.47 ± 0 *	0.44 ± 0.11 *	0.55 ± 0.04	0.13 ± 0.01	0.57 ± 0.04 *	6.50 ± 0.08 *
It39	0.13 ± 0.03	218.91 ± 0.03	13.20 ± 0.10	0.47 ± 0 *	0.37 ± 0 *	0.52 ± 0.07	0.24 ± 0.04	0.26 ± 0.08	6.12 ± 0.37
Lt2	0.24 ± 0.06	218.93 ± 0.06	13.15 ± 0.11	0.47 ± 0 *	0.37 ± 0.10 *	0.33 ± 0 *	4.12 ± 0.06 *	0.16 ± 0.01	8.48 ± 0.02 *
Mp51	0.70 ± 0.42	218.48 ± 0.42	12.10 ± 0.20 *	0.44 ± 0.01 *	1.39 ± 0.18 *	0.80 ± 0.05	0.14 ± 0.02	0.20 ± 0.01	5.83 ± 0.31
Mp FA	0.75 ± 0.35	218.29 ± 0.35	12.74 ± 0.28 *	0.46 ± 0.01 *	0.74 ± 0.26 *	0.70 ± 0.02	0.25 ± 0.02	0.12 ± 0.02 *	6.71 ± 0.33 *
Sl35	0.65 ± 0.52	218.39 ± 0.52	12.97 ± 0.15 *	0.47 ± 0 *	0.51 ± 0.12 *	0.72 ± 0.10	0.25 ± 0.06	0.20 ± 0.06	6.18 ± 0.08
Td35	0.87 ± 0.48	218.17 ± 0.48	13.12 ± 0.25 *	0.47 ± 0.01 *	0.34 ± 0.28 *	0.56 ± 0	0.33 ± 0.14 *	0.26 ± 0.01	5.14 ± 0.33
Td Pt	1.01 ± 0.04	218.03 ± 0.04	12.62 ± 0.06 *	0.46 ± 0 *	0.84 ± 0.06 *	0.95 ± 0.43	0.50 ± 0.01 *	0.31 ± 0.09	6.37 ± 0.61 *
Zb43	0.37 ± 0.50	218.80 ± 0.50	12.61 ± 0.21 *	0.45 ± 0 *	1.02 ± 0.05 *	0.83 ± 0.13	0.07 ± 0.04	0.30 ± 0.04	4.67 ± 0.09 *

Values are mean ± standard deviation of three independent replicates; The initial sugar concentration of synthetic and natural must was 200 and 219.2 g/L, respectively; * means statistically significant differences from the control sample in the same column (LSD test, *p* < 0.05).

## Supplementary Materials

The following are available online at https://www.mdpi.com/2076-2607/8/5/658/s1, Table S1: Glucose consumption (g/L), ethanol production (%, v/v) and ethanol yield (g/g) after 3 d in 5 mL YPD medium, Table S2: Analysis of sugars, ethanol, organic acids and glycerol from samples after 48 h of single fermentation.
